# Occurrence dataset of birds in the Xinjizhou National Wetland Park, Nanjing, China

**DOI:** 10.3897/BDJ.11.e103497

**Published:** 2023-05-15

**Authors:** Wei Shen, Zixi Zhao, Zheping Xu, Yong Zhang

**Affiliations:** 1 Co-Innovation Center for Sustainable Forestry in Southern China, College of Biology and the Environment, Nanjing Forestry University, Nanjing, China Co-Innovation Center for Sustainable Forestry in Southern China, College of Biology and the Environment, Nanjing Forestry University Nanjing China; 2 Chinese Academy of Sciences, Beijing, China Chinese Academy of Sciences Beijing China

**Keywords:** Xinjizhou National Wetland Park, wetland, birds, dataset

## Abstract

**Background:**

Xinjizhou National Wetland Park is located in Jiangning District, Nanjing, Jiangsu, China. With diverse wetland landscape types, Xinjizhou National Wetland Park maintains high biodiversity all year around. Meanwhile, as an important hub on the East Asian-Australasian Flyway, Xinjizhou National Wetland Park also occupies a core ecological position in the middle and lower reaches of the Yangtze River in Jiangsu Province. Therefore, carrying out systematic bird surveys and consequently understanding the distribution of birds and the seasonal variation of their communities can provide important insights for conservation. We conducted a one-year bird survey in the Xinjizhou National Wetland Park from 2021 to 2022 and provided occurrence datasets, including detailed species and geographic information.This dataset fills the knowledge gap in avian community composition for the Wetland Park and more importantly provides a basis to assess the conservation effectiveness of conservation measures taken in the Wetland Park.

**New information:**

This occurrence dataset is the first public record of birds in Xinjizhou National Wetland Park. All data have been published on GBIF.

## Introduction

Wetlands have a crucial ecosystem function and play a key role in maintaining the survival and reproduction of many organisms (Zedler and Kercher 2005). Birds are rich in species, widely distributed and easy to observe, their population dynamics, community structure and diversity are often used as important indicators for wetland environmental dynamic monitoring (Bart 2005, Zhou et al. 2019, Wang et al. 2022). Wetland parks are important type of constructed wetlands. As a supplement to natural wetlands, wetland parks organically integrate wetland protection, ecological restoration and sustainable utilisation of wetland resources and play an increasingly important role in global ecological protection and sustainable development (Wu et al. 2015).

Xinjizhou National Wetland Park is located in Jiangning District, Nanjing City. It lies in the upstream of the Jiangsu section of the Yangtze River, mainly including Xinji zhou, Xinsheng zhou, Zaisheng zhou, Zimu zhou and Zihui zhou. Xinjizhou National Wetland Park is an important representative of seasonally flooded wetlands in the middle and lower reaches of the Yangtze River. With diverse wetland landscape types, the Park maintains high biodiversity all the year round and, hence, is an important node for biodiversity conservation in the Basin (Wang et al. 2006, Cao et al. 2021).

At the same time, as an important transit station in the East Asian-Australasian Flyway (EAAF), it also occupies an important ecological position in Jiangsu and even the middle and lower reaches of the Yangtze River (Yong et al. 2015). Therefore, carrying out systematic bird surveys and consequently understanding the distribution of birds and the seasonal variation of their communities can provide important insights for conservation of regional bird resources (Ellis et al. 2019, Chen et al. 2021).

In this study, a systematic bird census was carried out monthly in Xinjizhou National Wetland Park from March 2021 to February 2022 to collate the bird distribution and community composition in the Wetland Park. Based on the annual monitoring results, the bird diversity and the seasonal variation of its community are analysed. The results can provide a scientific basis for the protection of bird diversity in Xinjizhou National Wetland Park and a detailed reference for the management and protection of beach wetlands in the middle and lower reaches of the Yangtze River.

## Sampling methods

### Sampling description

**Figure 1.**
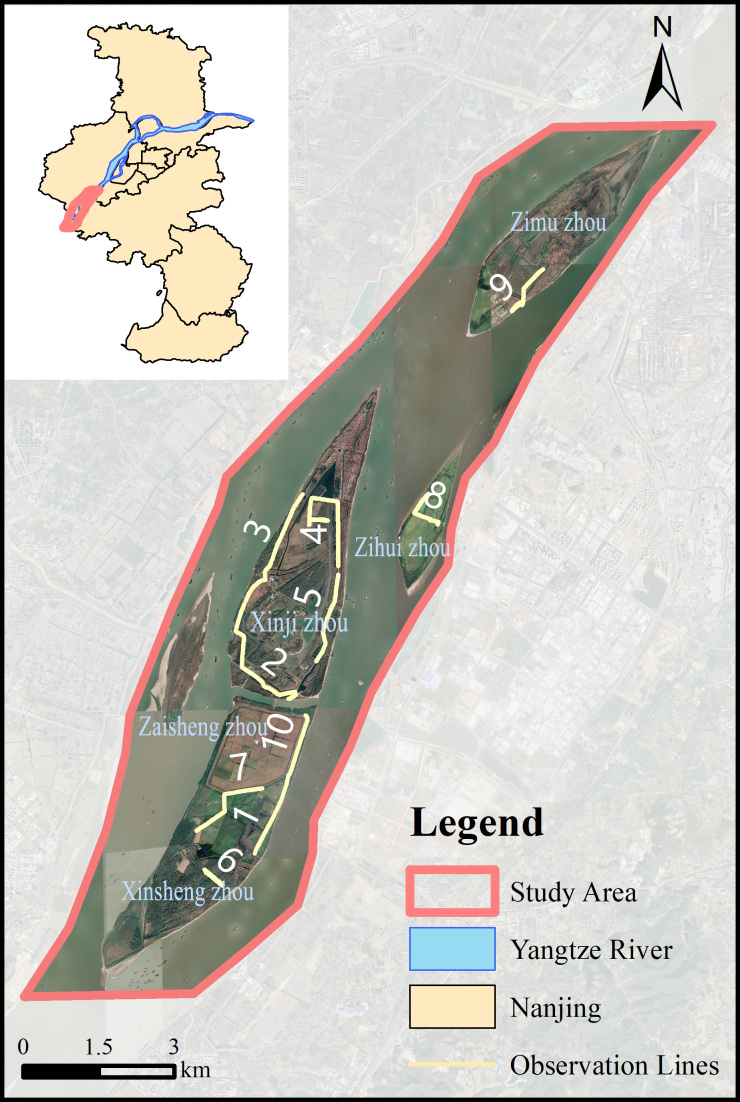
The location of the study area and the name of each island.

A total of 10 survey transects were set up in Xinjizhou National Wetland Park according to the beach area, habitat types and accessibility. The length of the transect ranged from 1.0 to 3.3 km, covering all habitat types in the Wetland Park (Fig. 1). From March 2021 to February 2022, a monthly bird survey was carried out on each of the 10 lines with binoculars (Shuntu 339FT 10*42) and monoculars (Swarovski ATS 20*60), recording birds species and their abundances. For species which were difficult to identify in the field, we used a camera (Canon Eos R6) with telephoto lens (Sigma 150-600mm f/5-6.3 DG OS HSM C) to take pictures and identify them later in the lab. The classification of birds was based on *A Checklist on the Classification and Distribution of the Birds of China* (*Third Edition*) (Zheng 2017). The final dataset was organised according to the Darwin Core format and uploaded to GBIF upon the conclusion of annual survey (Shen et al. 2023).

## Geographic coverage

### Description

We downloaded the Landsat8 satellite image and drew the investigation scope by using ArcGIS (10.7). A total of 10 survey lines were set up, with length between 1.0~3.3 km, covering all habitat types of Wetland Park, including four lines in Xinji Zhou (No. 2, 3, 4, 5), two lines in Xinsheng Zhou (No. 1, 6), two lines in Zaisheng Zhou (No. 7, 10), one line in Zimu Zhou (No. 9) and one line in Zihui Zhou (No.8) (Table 1).

### Coordinates

31.78 N and 31.93 N Latitude; 118.48 E and 118.59 E Longitude.

## Taxonomic coverage

### Description

A total of 42,542 birds were recorded in this occurrence dataset, belonging to 162 species, 47 families and 16 orders (Table 2). Most species are listed in the Red List of China's Vertebrates and the IUCN Red List (Jiang et al. 2016, IUCN 2022). In the Red List of China's Vertebrates, *Ciconiaboyciana* (Swinhoe, 1873) was ranked as National First-class Protected Wildlife; *Anseralbifrons* (Scopoli, 1769), *Cygnuscolumbianus* (Ord, 1815), *Aixgalericulata* (Linnaeus, 1758), *Nettapuscoromandelianus* (Gmelin, 1789), *Sibirionettaformosa* (Georgi, 1775), *Centropusbengalensis* (Gmelin, 1788), *Podicepsauritus* (Linnaeus, 1758), *Hydrophasianuschirurgus* (Scopoli, 1786), *Platalealeucorodia* (Linnaeus, 1758), *Elanuscaeruleus* (Desfontaines, 1789), *Accipitertrivirgatus* (Temminck, 1824), *Accipitersoloensis* (Horsfield, 1982), *Accipiternisus* (Linnaeus, 1758), *Accipitergentilis* (Linnaeus, 1758), *Milvusmigrans* (Boddaert, 1783), *Buteojaponicus* (Temminck & Schlegel, 1844), *Halcyonsmyrnensis* (Linnaeus, 1758), *Falcotinnunculus* (Linnaeus, 1758), *Falcosubbuteo* (Linnaeus, 1758), *Falcoperegrinus* (Tunstall, 1771), *Alaudaarvensis* (Linnaeus, 1758) and *Garrulaxcanorus* (Linnaeus, 1758) were ranked as National Second-class Protected Wildlife. In the IUCN Red List, *Marecafalcata* (Georgi, 1775), *Aythyanyroca* (Güldenstädt, 1770) and *Vanellusvanellus* (Linnaeus, 1758) were ranked as Near Threatened (NT); *Aythyaferina* (Linnaeus, 1758), *Podicepsnigricollis* (Brehm, 1831) and *Emberizarustica* (Pallas, 1776) were ranked as Vulnerable (VU); *Ciconiaboyciana* (Swinhoe, 1873) was ranked as Endangered (EN).

## Temporal coverage

### Notes

We conducted this survey from March 2021 to February 2022. The specific dates of this period were: 23/03/2021; 24/03/2021; 21/04/2021; 25/04/2021; 24/05/2021; 25/05/2021; 24/06/2021; 25/06/2021; 14/07/2021; 15/07/2021; 25/08/2021; 26/08/2021; 28/09/2021; 13/10/2021; 26/10/2021; 18/11/2021; 19/11/2021; 21/12/2021; 22/12/2021; 11/01/2022; 12/01/2022; 22/02/2022; 23/02/2022 (Table 3).

## Usage licence

### Usage licence

Creative Commons Public Domain Waiver (CC-Zero)

## Data resources

### Data package title

Occurrence dataset of birds in the Xinjizhou National Wetland Park, Nanjing, China

### Resource link

https://www.gbif.org/dataset/e631a759-36c9-4c34-98c4-35f9d110a1f5#description

### Alternative identifiers

http://www.gbifchina.org.cn/resource?r=xinjizhou

### Number of data sets

1

### Data set 1.

#### Data set name

Occurrence dataset of birds in the Xinjizhou National Wetland Park, Nanjing, China

#### Data format

Darwin Core Archive format

#### Download URL

https://www.gbif.org/zh/occurrence/download?dataset_key=e631a759-36c9-4c34-98c4-35f9d110a1f5

#### Description

Our occurrence dataset contains 35 column labels. All occurrence records are georeferenced. Due to the limitations of the sample line method, the positions of all recorded birds are replaced by lines' ID.

**Data set 1.** 

Column label	Column description
eventID	An identifier for the set of information associated with an Event (something that occurs at a place and time).
samplingProtocol	The names of, references to, or descriptions of the methods or protocols used during an Event.
samplingEffort	The amount of effort expended during an Event.
sampleSizeValue	A numeric value for a measurement of the size (time duration, length, area or volume) of a sample in a sampling event.
sampleSizeUnit	The unit of measurement of the size (time duration, length, area or volume) of a sample in a sampling event.
year	The four-digit year in which the Event occurred, according to the Common Era Calendar.
eventDate	The date-time or interval during which an Event occurred. For occurrences, this is the date-time when the event was recorded. Not suitable for a time in a geological context.
eventTime	The time or interval during which an Event occurred.
country	The name of the country or major administrative unit in which the Location occurs.
countryCode	The standard code for the country in which the Location occurs.
stateProvince	The name of the next smaller administrative region than country (state, province, canton, department, region etc.) in which the Location occurs.
locality	The specific description of the place.
locationID	An identifier for the set of location information (data associated with dcterms:Location). May be a global unique identifier or an identifier specific to the dataset.
location	A spatial region or named place.
decimalLatitude	The geographic latitude (in decimal degrees, using the spatial reference system given in geodeticDatum) of the geographic centre of a Location. Positive values are north of the Equator, negative values are south of it. Legal values lie between -90 and 90, inclusive.
decimalLongitude	The geographic longitude (in decimal degrees, using the spatial reference system given in geodeticDatum) of the geographic centre of a Location. Positive values are east of the Greenwich Meridian, negative values are west of it. Legal values lie between -180 and 180, inclusive.
geodeticDatum	The ellipsoid, geodetic datum or spatial reference system (SRS) upon which the geographic coordinates given in decimalLatitude and decimalLongitude are based.
coordinateUncertaintyInMetres	The horizontal distance (in metres) from the given decimalLatitude and decimalLongitude describing the smallest circle containing the whole of the Location. Leave the value empty if the uncertainty is unknown, cannot be estimated or is not applicable (because there are no coordinates). Zero is not a valid value for this term.
type	The nature or genre of the resource.
ownerInstitutionCode	The name (or acronym) in use by the institution having ownership of the object(s) or information referred to in the record.
language	A language of the resource.
occurrenceID	An identifier for the Occurrence (as opposed to a particular digital record of the occurrence). In the absence of a persistent global unique identifier, construct one from a combination of identifiers in the record that will most closely make the occurrenceID globally unique.
basisOfRecord	The specific nature of the data record.
individualCount	The number of individuals present at the time of the Occurrence.
organismQuantity	A number or enumeration value for the quantity of organisms.
organismQuantityType	The type of quantification system used for the quantity of organisms.
occurrenceStatus	A statement about the presence or absence of a Taxon at a Location.
scientificName	The full scientific name, with authorship and date information, if known. When forming part of an Identification, this should be the name in the lowest level taxonomic rank that can be determined. This term should not contain identification qualifications, which should instead be supplied in the IdentificationQualifier term.
kingdom	The full scientific name of the kingdom in which the taxon is classified.
phylum	The full scientific name of the phylum or division in which the taxon is classified.
class	The full scientific name of the class in which the taxon is classified.
order	The full scientific name of the order in which the taxon is classified.
family	The full scientific name of the family in which the taxon is classified.
taxonRank	The taxonomic rank of the most specific name in the scientificName.
recordedBy	A person, group or organisation responsible for recording the original Occurrence.

## Figures and Tables

**Table 1.** Distribution of survey transects for bird monitoring in Xinzhou National Wetland Park.

**Line number**	**Line location**	**Starting coordinates**	**Ending coordinates**	**Habitat type**
1	Zaisheng Zhou	31.82 N, 118.52 E	31.80 N, 118.51 E	wetland, woodland
2	Xinji Zhou	31.83 N, 118.52 E	31.85 N, 118.51 E	wetland, woodland
3	Xinji Zhou	31.85 N, 118.51 E	31.87 N, 118.52 E	wetland, woodland
4	Xinji Zhou	31.86 N, 118.53 E	31.86 N, 118.52 E	woodland
5	Xinji Zhou	31.84 N, 118.53 E	31.85 N, 118.53 E	wetland, woodland
6	Xinsheng Zhou	31.80 N, 118.51 E	31.80 N, 118.50 E	wetland, woodland
7	Xinsheng Zhou	31.82 N, 118.51 E	31.81 N, 118.50 E	wetland, woodland, grassland
8	Zihui Zhou	31.86 N, 118.55 E	31.87 N, 118.55 E	wetland, reedbed
9	Zimu Zhou	31.90 N, 118.57 E	31.91 N, 118.58 E	wetland, reed swamp
10	Zaisheng Zhou	31.83 N, 118.52 E	31.82 N, 118.52 E	wetland, woodland, reed swamp

**Table 2.** Birds species list in the Xinjizhou National Wetland Park.

**ID**	**Order**	**Family**	**English name**	**Scientific name**	**Total number of species**	**Red List of China's Vertebrates**	**IUCN Red List category**	**Quantity proportion**
1	Accipitriformes	Accipitridae	Black-winged Kite	*Elanuscaeruleus*	4	LC	LC	0.01%
2	Accipitriformes	Accipitridae	Crested Goshawk	*Accipitertrivirgatus*	1	LC	LC	0.00%
3	Accipitriformes	Accipitridae	Chinese Sparrowhawk	*Accipitersoloensis*	2	LC	LC	0.00%
4	Accipitriformes	Accipitridae	Eurasian Sparrowhawk	*Accipiternisus*	4	LC	LC	0.01%
5	Accipitriformes	Accipitridae	Northern Goshawk	*Accipitergentilis*	1	NT	LC	0.00%
6	Accipitriformes	Accipitridae	Black Kite	*Milvusmigrans*	81	LC	LC	0.19%
7	Accipitriformes	Accipitridae	Eastern Buzzard	*Buteojaponicus*	5	NT	LC	0.01%
8	Anseriformes	Anatidae	Bean Goose	*Anserfabalis*	72	EN	LC	0.17%
9	Anseriformes	Anatidae	Greater White-fronted Goose	*Anseralbifrons*	9	NT	LC	0.02%
10	Anseriformes	Anatidae	Tundra Swan	*Cygnuscolumbianus*	20	LC	LC	0.05%
11	Anseriformes	Anatidae	Ruddy Shelduck	*Tadornaferruginea*	7	LC	LC	0.02%
12	Anseriformes	Anatidae	Mandarin Duck	*Aixgalericulata*	3	NT	LC	0.01%
13	Anseriformes	Anatidae	Asian Pygmy Goose	*Nettapuscoromandelianus*	7	LC	LC	0.02%
14	Anseriformes	Anatidae	Baikal Teal	*Sibirionettaformosa*	6810	LC	LC	16.01%
15	Anseriformes	Anatidae	Gadwall	*Marecastrepera*	104	LC	LC	0.24%
16	Anseriformes	Anatidae	Northern Shoveler	*Spatulaclypeata*	28	LC	LC	0.07%
17	Anseriformes	Anatidae	Falcated Duck	*Marecafalcata*	1011	LC	NT	2.38%
18	Anseriformes	Anatidae	Eurasian Wigeon	*Marecapenelope*	28	LC	LC	0.07%
19	Anseriformes	Anatidae	Eastern Spot-billed Duck	*Anaszonorhyncha*	6528	NT	LC	15.34%
20	Anseriformes	Anatidae	Mallard	*Anasplatyrhynchos*	4521	LC	LC	10.63%
21	Anseriformes	Anatidae	Northern Pintail	*Anasacuta*	276	LC	LC	0.65%
22	Anseriformes	Anatidae	Green-winged Teal	*Anascrecca*	648	LC	LC	1.52%
23	Anseriformes	Anatidae	Common Pochard	*Aythyaferina*	30	LC	VU	0.07%
24	Anseriformes	Anatidae	Ferruginous Duck	*Aythyanyroca*	8	LC	NT	0.02%
25	Bucerotiformes	Upupidae	Common Hoopoe	*Upupaepops*	45	LC	LC	0.11%
26	Charadriformes	Recurvirostridae	Black-winged Stilt	*Himantopushimantopus*	1	LC	LC	0.00%
27	Charadriformes	Recurvirostridae	Pied Avocet	*Recurvirostraavosetta*	120	LC	LC	0.28%
28	Charadriformes	Charadriidae	Northern Lapwing	*Vanellusvanellus*	444	LC	NT	1.04%
29	Charadriformes	Charadriidae	Grey-headed Lapwing	*Vanelluscinereus*	208	LC	LC	0.49%
30	Charadriformes	Charadriidae	Little Ringed Plover	*Charadriusdubius*	6	LC	LC	0.01%
31	Charadriformes	Charadriidae	Kentish Plover	*Charadriusalexandrinus*	1	LC	LC	0.00%
32	Charadriformes	Jacanidae	Pheasant-tailed Jacana	*Hydrophasianuschirurgus*	69	LC	LC	0.16%
33	Charadriformes	Scolopacidae	Common Snipe	*Gallinagogallinago*	9	LC	LC	0.02%
34	Charadriformes	Scolopacidae	Common Sandpiper	*Actitishypoleucos*	3	LC	LC	0.01%
35	Charadriformes	Scolopacidae	Green Sandpiper	*Tringaochropus*	24	LC	LC	0.06%
36	Charadriformes	Scolopacidae	Common Redshank	*Tringatotanus*	2	NT	LC	0.00%
37	Charadriformes	Scolopacidae	Marsh Sandpiper	*Tringastagnatilis*	1	LC	LC	0.00%
38	Charadriformes	Scolopacidae	Wood Sandpiper	*Tringaglareola*	1	LC	LC	0.00%
39	Charadriformes	Scolopacidae	Spotted Redshank	*Tringaerythropus*	184	LC	LC	0.43%
40	Charadriformes	Scolopacidae	Common Greenshank	*Tringanebularia*	24	LC	LC	0.06%
41	Charadriformes	Glareolidae	Oriental Pratincole	*Glareolamaldivarum*	20	LC	LC	0.05%
42	Charadriformes	Laridae	Black-headed Gull	*Chroicocephalusridibundus*	2	LC	LC	0.00%
43	Charadriformes	Laridae	Whiskered Tern	*Chlidoniashybrida*	15	LC	LC	0.04%
44	Ciconiiformes	Ciconiidae	Oriental Stork	*Ciconiaboyciana*	16	NT	EN	0.04%
45	Columbiformes	Columbidae	Oriental Turtle Dove	*Streptopeliaorientalis*	421	LC	LC	0.99%
46	Columbiformes	Columbidae	Eurasian Collared Dove	*Streptopeliadecaocto*	4	LC	LC	0.01%
47	Columbiformes	Columbidae	Red Turtle Dove	*Streptopeliatranquebarica*	19	LC	LC	0.04%
48	Columbiformes	Columbidae	Spotted Dove	*Streptopeliachinensis*	502	LC	LC	1.18%
49	Columbiformes	Alcedinidae	Common Kingfisher	*Alcedoatthis*	20	LC	LC	0.05%
50	Columbiformes	Alcedinidae	Crested Kingfisher	*Megacerylelugubris*	1	LC	LC	0.00%
51	Columbiformes	Alcedinidae	Pied Kingfisher	*Cerylerudis*	64	LC	LC	0.15%
52	Columbiformes	Alcedinidae	White-throated Kingfisher	*Halcyonsmyrnensis*	3	LC	LC	0.01%
53	Cuculiformes	Cuculidae	Lesser Coucal	*Centropusbengalensis*	8	LC	LC	0.02%
54	Cuculiformes	Cuculidae	Chestnut-winged Cuckoo	*Clamatorcoromandus*	2	LC	LC	0.00%
55	Cuculiformes	Cuculidae	Common Koel	*Eudynamysscolopaceus*	17	LC	LC	0.04%
56	Cuculiformes	Cuculidae	Large Hawk Cuckoo	*Hierococcyxsparverioides*	16	EN	LC	0.04%
57	Cuculiformes	Cuculidae	Indian Cuckoo	*Cuculusmicropterus*	15	LC	LC	0.04%
58	Cuculiformes	Cuculidae	Common Cuckoo	*Cuculuscanorus*	9	NT	LC	0.02%
59	Falconiformes	Falconidae	Common Kestrel	*Falcotinnunculus*	2	LC	LC	0.00%
60	Falconiformes	Falconidae	Eurasian Hobby	*Falcosubbuteo*	3	LC	LC	0.01%
61	Falconiformes	Falconidae	Peregrine Falcon	*Falcoperegrinus*	2	LC	LC	0.00%
62	Galliformes	Phasianidae	Chinese Bamboo Partridge	*Bambusicolathoracicus*	25	LC	LC	0.06%
63	Galliformes	Phasianidae	Common Pheasant	*Phasianuscolchicus*	339	LC	LC	0.80%
64	Galliformes	Rallidae	Brown-cheeked Rail	*Rallusindicus*	3	LC	LC	0.01%
65	Galliformes	Rallidae	Brown Crake	*Zaporniaakool*	3	LC	LC	0.01%
66	Galliformes	Rallidae	White-breasted Waterhen	*Amaurornisphoenicurus*	4	LC	LC	0.01%
67	Galliformes	Rallidae	Common Moorhen	*Gallinulachloropus*	393	LC	LC	0.92%
68	Galliformes	Rallidae	Common Coot	*Fulicaatra*	510	LC	LC	1.20%
69	Passeriformes	Campephagidae	Ashy Minivet	*Pericrocotusdivaricatus*	2	LC	LC	0.00%
70	Passeriformes	Campephagidae	Swinhoe's Minivet	*Pericrocotuscantonensis*	18	LC	LC	0.04%
71	Passeriformes	Campephagidae	Black-winged Cuckoo-shrike	*Lalagemelaschistos*	18	NT	LC	0.04%
72	Passeriformes	Laniidae	Brown Shrike	*Laniuscristatus*	66	NT	LC	0.16%
73	Passeriformes	Laniidae	Long-tailed Shrike	*Laniusschach*	175	LC	LC	0.41%
74	Passeriformes	Oriolidae	Black-naped Oriole	*Orioluschinensis*	58	NT	LC	0.14%
75	Passeriformes	Dicruridae	Black Drongo	*Dicrurusmacrocercus*	170	LC	LC	0.40%
76	Passeriformes	Dicruridae	Ashy Drongo	*Dicrurusleucophaeus*	7	LC	LC	0.02%
77	Passeriformes	Monarchidae	Oriental Paradise Flycatcher	*Terpsiphoneaffinis*	8	LC	LC	0.02%
78	Passeriformes	Corvidae	Azure-winged Magpie	*Cyanopicacyanus*	458	LC	LC	1.08%
79	Passeriformes	Corvidae	Red-billed Blue Magpie	*Urocissaerythroryncha*	29	LC	LC	0.07%
80	Passeriformes	Corvidae	Grey Treepie	*Dendrocittaformosae*	316	LC	LC	0.74%
81	Passeriformes	Corvidae	Common Magpie	*Picapica*	874	LC	LC	2.05%
82	Passeriformes	Paridae	Great Tit	*Parusmajor*	535	LC	LC	1.26%
83	Passeriformes	Remizidae	Chinese Penduline Tit	*Remizconsobrinus*	70	LC	LC	0.16%
84	Passeriformes	Alaudidae	Oriental Skylark	*Alaudagulgula*	33	LC	LC	0.08%
85	Passeriformes	Alaudidae	Eurasian Skylark	*Alaudaarvensis*	1	LC	LC	0.00%
86	Passeriformes	Pycnonotidae	Collared Finchbill	*Spizixossemitorques*	10	LC	LC	0.02%
87	Passeriformes	Pycnonotidae	Light-vented Bulbul	*Pycnonotussinensis*	2693	LC	LC	6.33%
88	Passeriformes	Pycnonotidae	Black Bulbul	*Hypsipetesleucocephalus*	11	LC	LC	0.03%
89	Passeriformes	Hirundinidae	Barn Swallow	*Hirundorustica*	342	NT	LC	0.80%
90	Passeriformes	Hirundinidae	Red-rumped Swallow	*Cecropisdaurica*	142	LC	LC	0.33%
91	Passeriformes	Cettiidae	Rufous-faced Warbler	*Abroscopusalbogularis*	6	LC	LC	0.01%
92	Passeriformes	Cettiidae	Manchurian Bush Warbler	*Hororniscanturians*	56	LC	LC	0.13%
93	Passeriformes	Cettiidae	Brownish-flanked Bush Warbler	*Horornisfortipes*	39	LC	LC	0.09%
94	Passeriformes	Aegithalidae	Silver-throated Bushtit	*Aegithalosglaucogularis*	326	LC	LC	0.77%
95	Passeriformes	Aegithalidae	Black-throated Bushtit	*Aegithalosconcinnus*	66	LC	LC	0.16%
96	Passeriformes	Phylloscopidae	Yellow-browed Warbler	*Phylloscopusinornatus*	89	LC	LC	0.21%
97	Passeriformes	Phylloscopidae	Pallas's Leaf Warbler	*Phylloscopusproregulus*	65	LC	LC	0.15%
98	Passeriformes	Phylloscopidae	Dusky Warbler	*Phylloscopusfuscatus*	35	NT	LC	0.08%
99	Passeriformes	Acrocephalidae	Oriental Reed Warbler	*Acrocephalusorientalis*	3	LC	LC	0.01%
100	Passeriformes	Cisticolidae	Zitting Cisticola	*Cisticolajuncidis*	17	LC	LC	0.04%
101	Passeriformes	Cisticolidae	Plain Prinia	*Priniainornata*	11	LC	LC	0.03%
102	Passeriformes	Sylviidae	Grey-headed Parrotbill	*Psittiparusgularis*	1	LC	LC	0.00%
103	Passeriformes	Leiothrichidae	Hwamei	*Garrulaxcanorus*	3	LC	LC	0.01%
104	Passeriformes	Leiothrichidae	Greater Necklaced Laughingthrush	*Garrulaxpectoralis*	7	LC	LC	0.02%
105	Passeriformes	Leiothrichidae	Masked Laughingthrush	*Garrulaxperspicillatus*	799	LC	LC	1.88%
106	Passeriformes	Sylviidae	Vinous-throated Parrotbill	*Sinosuthorawebbiana*	1546	LC	LC	3.63%
107	Passeriformes	Sturnidae	Crested Myna	*Acridotherescristatellus*	125	LC	LC	0.29%
108	Passeriformes	Sturnidae	Silky Starling	*Spodiopsarsericeus*	255	LC	LC	0.60%
109	Passeriformes	Sturnidae	White-cheeked Starling	*Spodiopsarcineraceus*	1031	LC	LC	2.42%
110	Passeriformes	Sturnidae	Black-collared Starling	*Gracupicanigricollis*	2	LC	LC	0.00%
111	Passeriformes	Turdidae	White's Thrush	*Zootheraaurea*	4	LC	LC	0.01%
112	Passeriformes	Turdidae	Grey-backed Thrush	*Turdushortulorum*	2	LC	LC	0.00%
113	Passeriformes	Turdidae	Japanese Thrush	*Turduscardis*	4	LC	LC	0.01%
114	Passeriformes	Turdidae	Chinese Blackbird	*Turdusmandarinus*	666	LC	LC	1.57%
115	Passeriformes	Turdidae	Naumann's Thrush	*Turdusnaumanni*	5	LC	LC	0.01%
116	Passeriformes	Turdidae	Dusky Thrush	*Turduseunomus*	3	LC	LC	0.01%
117	Passeriformes	Muscicapidae	Oriental Magpie Robin	*Copsychussaularis*	6	LC	LC	0.01%
118	Passeriformes	Muscicapidae	Grey-streaked Flycatcher	*Muscicapagriseisticta*	1	LC	LC	0.00%
119	Passeriformes	Muscicapidae	Orange-flanked Bluetail	*Tarsigercyanurus*	34	LC	LC	0.08%
120	Passeriformes	Muscicapidae	Blue Whistling Thrush	*Myophonuscaeruleus*	6	LC	LC	0.01%
121	Passeriformes	Muscicapidae	Mugimaki Flycatcher	*Ficedulamugimaki*	1	LC	LC	0.00%
122	Passeriformes	Muscicapidae	Daurian Redstart	*Phoenicurusauroreus*	163	LC	LC	0.38%
123	Passeriformes	Muscicapidae	Siberian Stonechat	*Saxicolamaurus*	14	LC	LC	0.03%
124	Passeriformes	Passeridae	Eurasian Tree Sparrow	*Passermontanus*	403	NT	LC	0.95%
125	Passeriformes	Estrildidae	White-rumped Munia	*Lonchurastriata*	23	LC	LC	0.05%
126	Passeriformes	Motacillidae	Gray Wagtail	*Motacillacinerea*	1	LC	LC	0.00%
127	Passeriformes	Motacillidae	White Wagtail	*Motacillaalba*	89	LC	LC	0.21%
128	Passeriformes	Motacillidae	Olive-backed Pipit	*Anthushodgsoni*	181	LC	LC	0.43%
129	Passeriformes	Motacillidae	Buff-bellied Pipit	*Anthusrubescens*	1	LC	LC	0.00%
130	Passeriformes	Fringillidae	Brambling	*Fringillamontifringilla*	622	LC	LC	1.46%
131	Passeriformes	Fringillidae	Chinese Grosbeak	*Eophonamigratoria*	873	LC	LC	2.05%
132	Passeriformes	Fringillidae	Japanese Grosbeak	*Eophonapersonata*	43	LC	LC	0.10%
133	Passeriformes	Fringillidae	Grey-capped Greenfinch	*Chlorissinica*	143	LC	LC	0.34%
134	Passeriformes	Emberizidae	Tristram's Bunting	*Emberizatristrami*	1	LC	LC	0.00%
135	Passeriformes	Emberizidae	Little Bunting	*Emberizapusilla*	45	LC	LC	0.11%
136	Passeriformes	Emberizidae	Yellow-browed Bunting	*Emberizachrysophrys*	73	LC	LC	0.17%
137	Passeriformes	Emberizidae	Rustic Bunting	*Emberizarustica*	60	LC	VU	0.14%
138	Passeriformes	Emberizidae	Yellow-throated Bunting	*Emberizaelegans*	228	LC	LC	0.54%
139	Passeriformes	Emberizidae	Black-faced Bunting	*Emberizaspodocephala*	216	LC	LC	0.51%
140	Passeriformes	Emberizidae	Pallas's Bunting	*Emberizapallasi*	35	LC	LC	0.08%
141	Passeriformes	Emberizidae	Reed Bunting	*Emberizaschoeniclus*	20	LC	LC	0.05%
142	Pelecaniformes	Threskiornithidae	Eurasian Spoonbill	*Platalealeucorodia*	272	LC	LC	0.64%
143	Pelecaniformes	Ardeidae	Eurasian Bittern	*Botaurusstellaris*	2	LC	LC	0.00%
144	Pelecaniformes	Ardeidae	Yellow Bittern	*Ixobrychussinensis*	3	LC	LC	0.01%
145	Pelecaniformes	Ardeidae	Black Bittern	*Ixobrychusflavicollis*	4	LC	LC	0.01%
146	Pelecaniformes	Ardeidae	Black-crowned Night Heron	*Nycticoraxnycticorax*	261	LC	LC	0.61%
147	Pelecaniformes	Ardeidae	Striated Heron	*Butoridesstriata*	12	LC	LC	0.03%
148	Pelecaniformes	Ardeidae	Chinese Pond Heron	*Ardeolabacchus*	197	LC	LC	0.46%
149	Pelecaniformes	Ardeidae	Cattle Egret	*Bubulcusibis*	82	LC	LC	0.19%
150	Pelecaniformes	Ardeidae	Grey Heron	*Ardeacinerea*	402	LC	LC	0.94%
151	Pelecaniformes	Ardeidae	Purple Heron	*Ardeapurpurea*	11	LC	LC	0.03%
152	Pelecaniformes	Ardeidae	Great Egret	*Ardeaalba*	174	LC	LC	0.41%
153	Pelecaniformes	Ardeidae	Intermediate Egret	*Ardeaintermedia*	25	NT	LC	0.06%
154	Pelecaniformes	Ardeidae	Little Egret	*Egrettagarzetta*	364	LC	LC	0.86%
155	Piciformes	Picidae	Speckled Piculet	*Picumnusinnominatus*	4	NT	LC	0.01%
156	Piciformes	Picidae	Grey-capped Woodpecker	*Dendrocoposcanicapillus*	24	LC	LC	0.06%
157	Piciformes	Picidae	Great Spotted Woodpecker	*Dendrocoposmajor*	33	LC	LC	0.08%
158	Piciformes	Picidae	Grey-headed Woodpecker	*Picuscanus*	1	LC	LC	0.00%
159	Podicipediformes	Podicipedidae	Little Grebe	*Tachybaptusruficollis*	406	LC	LC	0.95%
160	Podicipediformes	Podicipedidae	Horned Grebe	*Podicepsauritus*	1	LC	VU	0.00%
161	Podicipediformes	Podicipedidae	Great Crested Grebe	*Podicepscristatus*	55	LC	LC	0.13%
162	Suliformes	Phalacrocoracidae	Great Cormorant	*Phalacrocoraxcarbo*	1937	LC	LC	4.55%

**Table 3.** Survey details

No.	Start Date	End Date	Sites Completed	Sites Not Completed
1	23/03/2021	24/03/2021	1 2 3 4 5 6 7 8 9 10	
2	21/04/2021	25/04/2021	1 2 3 4 5 6 7 8 9 10	
3	24/05/2021	25/05/2021	1 2 3 4 5 6 7 10	8 (due to high water level)
4	24/06/2021	25/06/2021	1 2 3 4 5 6 7 8 9 10	
5	14/07/2021	15/07/2021	1 2 3 4 5 6 7 8 9 10	
6	25/08/2021	26/08/2021	1 2 3 4 5 6 7 8 9 10	
7	28/09/2021	-	1 2 3 4 5 6 7 10	8 9 (due to high water level)
8	13/10/2021	26/10/2021	1 2 3 4 5 6 7 8 9 10	
9	18/11/2021	19/11/2021	1 2 3 4 5 6 7 8 9 10	
10	21/12/2021	22/12/2021	1 2 3 4 5 6 7 8 9 10	
11	11/01/2022	12/01/2022	1 2 3 4 5 6 7 8 9 10	
12	22/02/2022	23/02/2022	1 2 3 4 5 6 7 8 9 10	
